# The effect of umbilical cord cleansing with chlorhexidine gel on neonatal mortality among the community births in South Sudan: a quasi-experimental study

**DOI:** 10.11604/pamj.2021.38.78.21713

**Published:** 2021-01-22

**Authors:** Christopher Vunni Draiko, Kevin McKague, Judith Draleru Maturu, Sitima Joyce

**Affiliations:** 1 Better Health Care Organization, Juba, South Sudan,; 2 Canada Research Chair in Social Enterprise and Inclusive Markets, Cape Breton University, Sydney, Nova Scotia, Canada

**Keywords:** Newborn, mortality, infections, chlorhexidine, safe delivery kit

## Abstract

**Introduction:**

the use of chlorhexidine antiseptic gel for umbilical cord care in unhygienic settings has been shown to reduce infection and neonatal mortality in Asia, leading to the revision of WHO guidelines. However, few studies exist in the African context and none have been undertaken in conflict-affected settings. We aimed to assess the effectiveness of applying chlorhexidine gel to the umbilical cord stump on cord sepsis and neonatal mortality rates in the Republic of South Sudan.

**Methods:**

our pre/post quasi-experimental study recruited 3,143 pregnant women from six rural communities in Jubek County, South Sudan: 1,825 women in the treatment group and 1,318 women in the control group. Neonates in the treatment group had chlorhexidine applied to the umbilical cord stump within 24 hours of birth and daily for seven days. No chlorhexidine gel was applied in the control group, instead they were encouraged to practice dry cord care. Data was collected at enrolment and at each antenatal visit at 3, 7, 14 and 28 days. Our primary outcomes of interest were incidence of neonatal umbilical cord sepsis and neonatal mortality, which were analyzed on an intention-to-treat basis. The study is registered with Pan African Clinical Trial Registry, Number PACTR201808694484456.

**Results:**

the neonatal cord infection rate among the treatment group was 17.0%, compared to 38.9% in the control group (P<0.05), which was statistically significant. Neonatal mortality was least in the intervention (1.3%) and highest in the control (13.3%) group, which was also statistically significant.

**Conclusion:**

our evidence showed that chlorhexidine gel application contributed to the reduction of cord sepsis and neonatal mortality in conflict-affected South Sudan where the majority of births happen at home in unsanitary conditions. Chlorhexidine gel should be added to the essential medicines list in South Sudan and a costed plan for scale-up of chlorhexidine gel application should be developed by the Ministry of Health.

## Introduction

Of the 2.6 million annual neonatal deaths worldwide, [1] 99% occur in low- and middle-income countries and three-quarters occur in the first week of life [2]. Globally, one third of neonatal deaths are attributed to infections [2]. The greatest risk of infection is during home delivery in unhygienic conditions with under-skilled birth attendants and sub-optimal delivery conditions and practices [3]. The highest neonatal mortality rates occur in sub-Saharan Africa with conflict-affected South Sudan having one of the highest neonatal mortality rates in the world at 40 per 1,000 live births [4]. In South Sudan, 64% of neonatal deaths are caused by infection, and sepsis from the infected umbilical cord is one of the main pathways leading to neonatal death [5]. Approximately 80% of women give birth at home [5] with 93% of houses in the country being grass thatched mud huts [6]. Applications of unsafe substances to the newly cut umbilical cord increase risk of neonatal sepsis and mortality [7]. Based on studies in Nepal [8], Pakistan [9] and Bangladesh [10], the World Health Organization recommended the use of chlorhexidine antiseptic gel for cord care for home births in contexts like South Sudan where home conditions are unhygienic and neonatal mortality rates are higher than 30 per 1,000 live births [11]. However, few studies have examined the effectiveness of chlorhexidine for umbilical cord care in sub-Saharan Africa and none have examined it in a conflict-affected context. Our study addresses this gap by assessing the effect of umbilical cord cleansing with chlorhexidine gel with dry cord care (the WHO standard in the absence of chlorhexidine).

## Methods

**Study design and community selection:** our quasi-experimental study was conducted in six rural communities in Jubek County, South Sudan. Each community was defined as those living within a four-hour walk of a government primary health care facility. The six communities were purposively selected in partnership with South Sudan Ministry of Health officials at federal and state levels to ensure that they had a previously trained health worker active in the community that could assist with data gathering for the study. The communities were otherwise deemed to be representative of communities in Jubek County. The three treatment communities and three control communities were selected to be suitably distant from each other to avoid the possibility of contamination. Enrolled mothers in the three treatment communities would receive chlorhexidine to apply to the umbilical cord stump and enrolled mothers in the control communities would practice dry cord care.

**Sample size:** calculation of sample size was based on the ability to detect a 20% decrease in the neonatal mortality due to umbilical cord infections and 75% decrease in the intervention group. For sample with an error of 0.05, 20% drop rate. The value was used as references. Using G* power version to determine the sample size of 300 pregnant mother in each including the lost to follow up. However, the actual sample size of the mother were two folds in both group.

**Trial background:** the study was approved by the Institutional Review Board of the Directorate of Policy, Research, Planning and Budgeting for the Ministry of Health of the Republic of South Sudan. Field Data collection was approved by the Ministry of Health in Jubek State in South Sudan and through the national Directorate of Reproductive Health Service. The trial is registered with Pan African Clinical Registry with registration number PACTR201808694484456.

**Preliminary investigations:** we began our investigation with a preliminary survey and data gathering process in Jubek County to gain a greater understanding of local cord care practices and the optimal ways to make chlorhexidine gel available in this setting. A preliminary survey of 300 mothers reported that only 4% had practiced dry cord care. Instead, after cutting the umbilical cord, respondents reported that 48% applied ash, 39% applied water with salt, 22% applied the powder from a burnt match stick, 17% applied a milky fluid from the Leben-Leben tree, 13% applied soil mixed with oil and 9% applied a combination of herbs (with multiple methods used). We also interviewed Ministry of Health officials at Federal and State levels and local health workers in Jubek County to understand the most effective way to make chlorhexidine available to mothers and health workers. Based on our interviews, we concluded that bundling tubes of chlorhexidine gel in Safe Delivery Kits was the best way to make chlorhexidine accessible to mothers and health workers.

**Health worker training and community awareness:** a total of 86 health workers (including traditional birth assistants) in the six study communities were trained for two days. Health workers in the three treatment communities were trained on chlorhexidine gel application (chlorhexidine digluconate 7.1% delivering 4.1% gel to the umbilical cord stump), how to screen and enroll mothers into the study and in undertaking regular follow-up visits and documentation. Health workers in the intervention group were trained on dry cord care, how to screen and enroll mothers into the study, and in undertaking regular follow-up visits and documentation. We also delivered general public awareness activities in the study communities that focused on the importance of prenatal and antenatal care and mothers giving birth with the help of a skilled health worker.

### Participant enrolment

Beginning in May 2018, pregnant women in one of the three treatment or three control communities who met the following eligibility criteria were enrolled in the study: they were in their second or third trimester; fifteen years or older; planning to stay in their community at least 28 days after delivery; and willing to provide cord care according to the relevant protocol. Pregnant women were identified by health workers or field research team members at health facilities, community meetings or through other contacts in the community. Recruited women provided verbal consent for their participation. The research team comprised of two field supervisors and a data quality monitoring officer. The study team worked with the trained community health workers who monitored and visited the enrolled pregnant mothers at their homes. During initial enrolment, data was gathered on the women´s number of children, expected date of delivery, health status and demographic characteristics such as name, location, age, and educational level. Enrolled women were also given standard prenatal information and messages by health workers including the value of giving birth with a trained health worker or birth attendant and the importance of breastfeeding. The study team and health workers developed a communication system to notify each other within 24 hours of an enrolled woman giving birth. Before delivery, during prenatal visits by a health worker, pregnant mothers in the intervention group received a safe delivery kit with chlorhexidine bundled with the regular contents of soap, a sterile razor blade, sterile gloves, two cord clamps, candle and matches and a plastic mat. The safe delivery kit for those in the control group were the same but without the chlorhexidine gel. Mothers in the treatment group were trained on how to apply the chlorhexidine gel to the umbilical cord immediately after it is cut and then daily for seven days. Mothers in the control group were reminded of the importance of dry cord care and not applying any substances to the cut umbilical cord. After delivery, the mother and newborn were visited by the community health workers and field supervisor on days 0, 3, 7, 14 and 28. At each visit, information on the newborns were collected including their sex, weight, health status, chlorhexidine gel applications, danger signs and sign of infections at the umbilical cord. The mother and neonate´s health were regularly assessed for vital signs and normality. In the chlorhexidine gel intervention group, the community health workers and field supervisor confirmed the application of chlorhexidine gel, and in the control group they asked whether other substances were applied to the cord or if the cord remained dry. In the treatment group, the field supervisor documented the time of chlorhexidine gel application and mothers were asked about the availability of chlorhexidine and where necessary provided with additional packages. The mothers were advised to keep the empty packs of the chlorhexidine as evidence of application for collection during the follow-up visits.

The team reviewed a list of questions with the mother at each visit about her health status and that of newborn. If any signs of cord infection were found present, the newborn was referred to the nearest health center for evaluation. After the day 28 home visit was completed, the project lead and field supervisor for each of the project locations completed the information on the mother and her newborn. Data was collected using paper forms designed by the study team. Field supervisors and community health workers met every week with the field supervisors to submit the data collected using the paper-based form. Field supervisors reviewed the submitted data for completeness, consistency, errors and missing data, where errors or missing data were noted the field team was asked to fix and follow-up on missing data where possible. Every week, the hard copy of the data was transmitted to the office and was securely stored for future reference. The field supervisor reviewed forms for accuracy and completeness, and brought the forms to the data officer, where data was entered into an Excel spreadsheet and exported into SPSS version 22. Where data was found missing, inconsistent or inaccurate, the data officer conducted field visits to verify and conduct data quality audits with mothers and health workers. The study team also conducted regular visits to the nearest health facility and collected information on admission of any referred mothers and neonates with various morbidities. The information was collected continuously from the health centers located within the intervention and control areas. The field supervisor conducted a verbal autopsy on a neonate´s mortality whenever it was reported through the health facility or through the community communication system. The study field team interviewed the mothers, caretaker, family members and relatives to confirm mortality. Where the mother died during the process of giving birth, the husband or relatives were interviewed on the causes of the neonate´s death. The project team and the community health workers were taught to monitor any adverse effects related to application of chlorhexidine gel. In this study, we anticipated four potential types of adverse events related to chlorhexidine gel application, all of which we anticipated to occur very rarely, if at all. These included accidental ingestion of chlorhexidine, accidental application on the eye as ointment (ocular exposure), local skin irritation around the umbilical stump and anaphylaxis. During our intervention, there was no reported incidence of adverse effects related to chlorhexidine gel application.

**Outcome measures:** the primary outcomes were neonatal mortality and sepsis within 28 days´ post-partum among neonates who survived the first 24 hours after delivery. Deaths were documented by interview with the mother. Our study excluded still births and premature newborn and a newborn was considered still birth when the neonates did not did not breathe, cry, or move at the time of delivery. Information was recorded on the time of birth and death and surrounding circumstances. Secondary outcomes, reported were incidence of cord infections categorized into four levels of severity according redness, pus, swelling and discharge. Additional secondary outcomes included proportion of women who deliver home or health facility, adverse events were defined as chlorhexidine-related events including accidental ingestion, accidental ocular exposure, contact dermatitis, or skin irritation around the umbilical stump and anaphylaxis. The study group comprised the safety monitoring group and convened twice and discussed the study and reviewed progress. The study gathered data between May 1^st^2018 and February 28^th^2019.

**Data analysis:** our primary objective and analyses was to determine the effectiveness of chlorhexidine integrated into safe delivery kit. Newborn mortality and cord infections due to cord infections analysis among newborns who survived for 24 hours was conducted on an intent to treat analysis. We then compared these characteristics between the two groups that may be related to the outcome, on reasonably balanced communities, mothers and newborn. Categorical variables were compared between groups using chi square tests and continuous variables using t tests for normally distributed or non-parametric Wilcoxon rank sum tests, for non-normal distribution.

## Results

**Figure 1:**
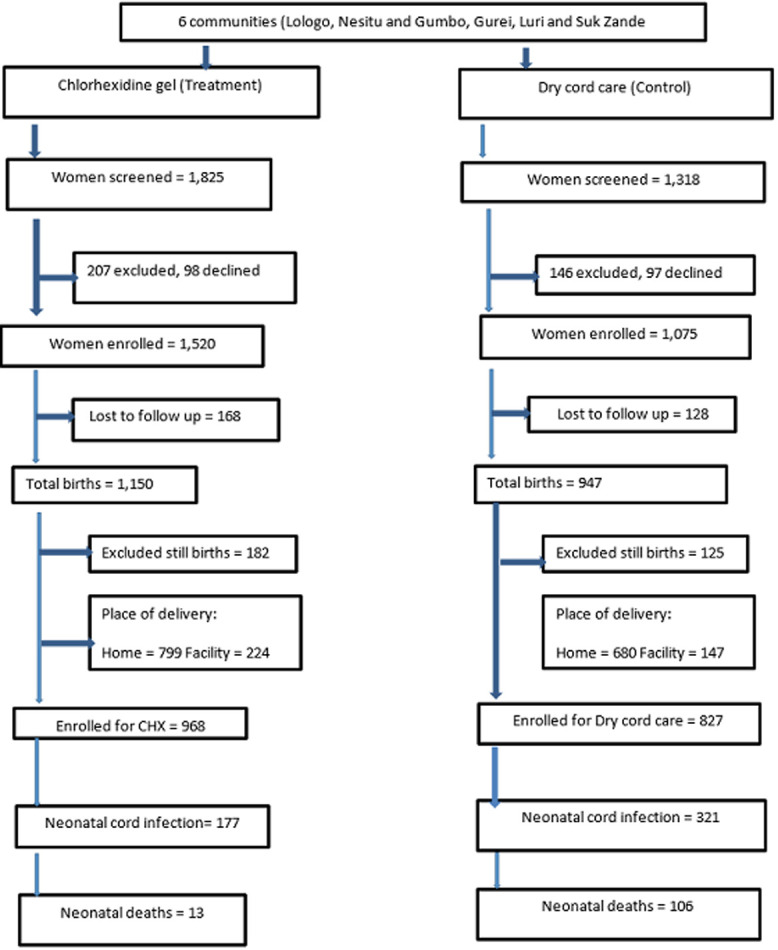
flow chart for recruitment

Of the 3,143 pregnant women screened for the study, 2,595 were eligible (1,520 in the treatment group and 1,075 in the control group) (Figure 1). A total of 397 pregnant mothers (168 interventions; 128 control) were lost to follow-up because they had relocated or withdrew based on the cultural norm that women should not be followed. A total of 2,650 women delivered (1,150 interventions; 947 control) with 40 set of twins, 307 still births and 2,096 live births who survived in the first 24 hours. Therefore 1,790 neonates were enrolled (968 for chlorhexidine cord care treatment and 822 for dry cord care control) Figure 1.

The comparison of socio-demographic indicators between treatment and control groups is summarized in Table 1. The majority of women were between 20-29 years of age, married and identified their occupation as house wives. Most had no formal education. There were no significant differences in their socio-demographic characteristics except more women were married in the intervention (96.5%) group than in the control (80.2%) group. However, we are not aware of any systematic biases in the selection and screening process that could have contributed to this difference.

**Table 1:** maternal demographic characteristics

		Group	
Variable		Intervention N (%)	Control N (%)
**Age in years**	15-19	281(27.5)	195(23.6)
	20-29	537(52.5)	430(52.0)
	30-39	204(19.9)	195(23.6)
	40+	1(0.1)	7(0.8)
**Marital status**	Married	987(96.5)	663(80.2)
	Single	18(1.8)	91(11.0)
	Separated	10(1.0)	34(4.1)
	Divorced	8(0.8)	39(4.7)
**Occupation**	House wife	678(66.3)	602(72.8)
	Farmer	299(29.2)	194(23.5)
	Businesswoman	33(3.2)	22(2.7)
	Government worker	13(1.3)	9(1.1)
**Parity**	First child	36(3.5)	42(5.1)
	Two children	209(20.4)	190(23.0)
	Three children	86(8.4)	62(7.5)
	Four children	139(13.6)	123(14.9)
	Five children	200(19.6)	98(11.9)
	Six children	108(10.6)	138(16.7)
	Seven children	115(11.2)	91(11.0)
	Eight children	103(10.1)	69(8.3)
	More than nine children	27(2.6)	14(1.7)
**Education**	Primary	212(20.7)	169(20.4)
	Secondary	188(18.4)	160(19.3)
	Institutions	57(5.6)	46(5.6)
	No Formal education	566(55.3)	452(54.7)
**Trimester**	Second	552(54.1)	429(51.9)
	Third	470(45.9)	398(48.1)

**Antenatal care attendance, delivery and follow-up:** a majority of the pregnant women in both treatment and control groups attended at least two anti-natal visits. Most women gave birth at home (78.1% treatment; 82.2% control). The majority (99.1%) of neonates in the treatment group received chlorhexidine gel application within 24 hours. There was no chlorhexidine gel application reported in the control group. The majority of mothers and neonates in the treatment and control groups were followed up on days 3, 7, 14 and 28 (Table 2).

**Table 2:** maternal antenatal care attendance, place of delivery and follow-up

		Group			
Variables				COR (95%CI)	P- Value
**ANC services**		Intervention N (%)	Control N (%)		
**ANC 1**	No	99(9.7)	92(11.1)		
	Yes	924(90.3)	735(88.9)		
**ANC 2**	No	325(31.8)	299(36.2)		
	Yes	698(68.2)	528(63.8)		
**ANC 3**	No	579(56.6)	373(45.1)		
	Yes	444(43.4)	454(54.9)		
**ANC 4**	No	811(79.3)	774(93.6)		
	Yes	212(20.7)	53(6.4)		
**Place of delivery**	Health facility	224(21.9)	147(17.8)	1.29(1.03-1.64)	0.028**
	Home	799(78.1)	680(82.2)		
**Sex of the newborn**	Male	479(46.8)	359(43.4%		
	Female	544 (53.2)	468(56.6)		
**Chlorhexidine Gel**	24 Hour	1014(99.1)	-		
	After 24 Hours	9(0.9)	-		
**Follow-up day 3**	Yes	1023(100.0	827(100.0)		
	No	00(0.0)	00(0.0)		
**Follow-up day 7**	Yes	1005(98.2)	821(99.3)	2.45(0.97-6.20)	0.051
	No	18(1.8)	6(0.7)		
**Follow- up day 14**	Yes	957(93.5)	734(88.8)	0.54(0.39-076)	0.000**
	No	66(6.5)	93(11.2)		
**Follow-up day 28**	Yes	889(86.9)	684(82.7)	0.72(0.56-0.931)	0.012**
	No	134(13.1)	143(17.3)		

Significant level 0.05*

**Maternal and neonatal condition and referrals:** 8.5% of mothers in the treatment group and 11.4% in the control group had danger signs associated with pregnancy and delivery (Table 3). Meanwhile 13.1% of neonates in the treatment group exhibited danger signs compared to 27.4% in the control group. The majority of danger signs were associated with cord infections. The treatment group had a higher incidence of mild redness while the control group showed a higher incidence of severe redness, pus and pain at the umbilical cord. Both neonates and mothers who exhibited danger signs of infection were referred to health facilities for additional care.

**Table 3:** maternal and neonatal condition and referrals

		Group	
		Intervention N (%)	Control N (%)
Maternal danger signs	No	936(91.5)	733(88.6)
	Yes	87(8.5)	94(11.4)
Conditions	Mother feels hot (proxy for fever)	25(30.1)	38(44.2)
	Prolonged or excessive vaginal bleeding	7(8.4)	7(8.1)
	Headaches, blurred vision, swollen hands and face (pre-eclampsia)	20(24.1)	19(22.1)
	Convulsions, unconsciousness (eclampsia)	22(26.5)	6(7.0)
	Prolonged or difficult labor	3(3.6)	5(5.8)
	Fever and foul smelling vaginal discharge	6(7.2)	11(12.8)
Maternal referrals	Yes	86(8.4)	88(10.6)
	No	937(91.6)	739(89.4)
Neonatal danger signs	Yes	134(13.1)	227(27.4)
	No	889(86.9)	600(72.6)
Sign of cord infections	Mild redness	69(39.4)	115(32.8)
	Moderate redness	43(24.6)	85(24.2)
	Severe redness	16(9.1)	55(15.7)
	Presence of pus	6(3.4)	63(17.9)
	Pain at the cord	41(23.4)	33(9.4)
Neonatal referrals	Yes	201(19.6)	235(28.4)
	No	822(80.4)	592(71.6)

**Cord infections and neonatal deaths:** the neonatal cord infections in the treatment group was 17.3 % compared to 38.8% in the control group (Table 4). Neonatal mortality was least in the intervention (1.3%) and highest in the control (13.3%) group.

**Table 4:** cord infections and neonatal deaths

Variables		Interventions, N (%)	Control, N (%)	COR (95%CI)	P- Value
Cord infections	Yes	177 (17.3)	321(38.8)	3.03(2.45-3.76)	0.000**
	No	846 (82.7)	506(61.2)		
All neonatal deaths		13(1.3)	109(13.3)	2.16(1.10-4.22)	0.022**

Significant level 0.05**

**Funding:** the study received funding from Grand Challenges Canada (GCC) for small innovation

## Discussion

The study presented the results of piloting the use of chlorhexidine gel integrated into safe delivery kits in Jubek County, South Sudan. Evidence from our pilot study showed that chlorhexidine gel was effective in reducing neonatal cord infections and mortality compared to dry cord practices. The uptake and use of chlorhexidine in the treatment group among individuals that had previously been using unhygienic substances on the umbilical cord suggests that chlorhexidine use can be broadly accepted among mothers and community members. There were several strengths of the pilot study thus far that could be leveraged further. It was a culturally relevant, people-centered, public health approach and it served a clear, perceived need of the communities. Community trust was actively cultivated and leveraged in the upfront design exercise and throughout the execution of the study. The overall design approach involved all levels of community structures and the Ministry of Health at State and Federal levels. The distributions system for chlorhexidine-equipped safe delivery kits established by the study can be further extended throughout the country. Our intervention revealed that the packing of the chlorhexidine gel into a safe delivery kit and distributing it to community health workers or mothers before delivery promoted local ownership and use.

## Conclusion

Evidence from our study showed that application of chlorhexidine gel to the umbilical cord stump after cutting and during the first seven days reduced cord sepsis and neonatal mortality compared to control groups practicing dry cord care. These findings in conflict-affected South Sudan are consistent with previous studies in Asia. The findings suggest that the South Sudanese Ministry of Health add chlorhexidine to its list of essential medicines and develop a costed plan for a phased scale-up dissemination of chlorhexidine gel in safe delivery kits across the country, while developing a randomized trial to further validate the findings of this quasi-experimental pre-post study.

### What is known about this topic

- The few studies conducted on chlorhexidine gel cord cleansing in Africa demonstrated limited effects on reducing umbilical cord and reduced neonatal mortality among the community and hospital births;
- Studies in South East Asia demonstrated that chlorhexidine for cord cleansing was effective in reducing cord infections and neonatal mortality.

### What this study adds

- Chlorhexidine gel integrated into safe delivery kit for cord cleansing for home births was effective in reducing neonatal cord infections and mortality;
- There is increase uptake and use of chlorhexidine gel among the mothers for umbilical cord cleansing that was previously using unhygienic and rudimentary substances on the cord.

## References

[1] World Health Organization Global Health Observatory Data on Neonatal Mortality Situation and Trends.

[2] Lawn JE, Cousens S, Zupan J (2005). 4 million Newborn deaths: When?. Where? Why? Lancet.

[3] Mullany LC, Darmstadt GL, Katz J, Khatry SK, LeClerq SC, Tielsch JM (2006). Risk factors for umbilical cord infection among newborns of southern Nepal. American journal of epidemiology.

[4] Republic of South Sudan (2018). Reproductive, Maternal, Newborn, Child, Adolescent Health and Nutrition Strategic Plan 2019 to 2023.

[5] Republic of South Sudan (2018). National Scale-up of 7.1% Chlorhexidine Gel Integrated into Safe Delivery Kit for Reduction of Neonatal Mortality in South Sudan.

[6] Kayiira D (2012). South Sudan Housing Finance Report. Centre for Affordable Housing Finance in Africa.

[7] Thaver D, Zaidi AK Burden of neonatal infections in developing countries: a review of evidence from community-based studies. The Pediatric infectious disease journal. 2009;.

[8] Mullany LC, Darmstadt GL, Khatry SK, Katz J, LeClerq SC, Shrestha S (2006). Topical applications of chlorhexidine to the umbilical cord for prevention of omphalitis and neonatal mortality in southern Nepal: a community-based, cluster-randomised trial. The Lancet.

[9] Soofi S, Cousens S, Imdad A, Bhutto N, Ali N, Bhutta ZA Topical application of chlorhexidine to neonatal umbilical cords for prevention of omphalitis and neonatal mortality in a rural district of Pakistan: a community-based, cluster-randomised trial. The Lancet. 2012;.

[10] El Arifeen S, Mullany LC, Shah R, Mannan I, Rahman SM, Talukder MR The effect of cord cleansing with chlorhexidine on neonatal mortality in rural Bangladesh: a community-based, cluster-randomised trial. The Lancet. 2012;.

[11] World Health Organization (2014). WHO recommendations on postnatal care of the mother and newborn. World Health Organization.

